# Green composites made of polyhydroxybutyrate and long-chain fatty acid esterified microcrystalline cellulose from pineapple leaf

**DOI:** 10.1371/journal.pone.0282311

**Published:** 2023-03-03

**Authors:** Pitchanun Sinsukudomchai, Duangdao Aht-Ong, Kohsuke Honda, Suchada Chanprateep Napathorn

**Affiliations:** 1 Department of Microbiology, Faculty of Science, Chulalongkorn University, Patumwan, Bangkok, Thailand; 2 Department of Materials Science, Faculty of Science, Chulalongkorn University, Patumwan, Bangkok, Thailand; 3 Center of Excellence on Petrochemical and Materials Technology, Chulalongkorn University, Bangkok, Thailand; 4 International Center for Biotechnology, Osaka University, Suita, Osaka, Japan; Karl-Franzens-Universitat Graz, AUSTRIA

## Abstract

Pineapple leaf fibres are an abundant agricultural waste product that contains 26.9% cellulose. The objective of this study was to prepare fully degradable green biocomposites made of polyhydroxybutyrate (PHB) and microcrystalline cellulose from pineapple leaf fibres (PALF-MCC). To improve compatibility with PHB, the PALF-MCC was surface modified using lauroyl chloride as an esterifying agent. The influence of the esterified PALF-MCC laurate content and changes in the film surface morphology on biocomposite properties was studied. The thermal properties obtained by differential scanning calorimetry revealed a decrease in crystallinity for all biocomposites, with 100 wt% PHB displaying the highest values, whereas 100 wt% esterified PALF-MCC laurate showed no crystallinity. The addition of esterified PALF-MCC laurate increased the degradation temperature. The maximum tensile strength and elongation at break were exhibited when adding 5% of PALF-MCC. The results demonstrated that adding esterified PALF-MCC laurate as a filler in the biocomposite film could retain a pleasant value of tensile strength and elastic modulus whereas a slight increase in elongation can help to enhance flexibility. For soil burial testing, PHB/ esterified PALF-MCC laurate films with 5–20% (w/w) PALF-MCC laurate ester had higher degradation than films consisting of 100% PHB or 100% esterified PALF-MCC laurate. PHB and esterified PALF-MCC laurate derived from pineapple agricultural wastes are particularly suitable for the production of relatively low-cost biocomposite films that are 100% compostable in soil.

## Introduction

Petroleum-based plastics have been a major source of innovation-driven technologies due to their unique properties, including low price; therefore, they remain a promising material for future technologies. However, their high usage volume has resulted in the unrelenting rise in plastic and microplastic pollution, which calls for global policies and legislation [1, 2]. Over the past two decades, bioplastics have increasingly been promoted as a solution to the problems of conventional plastics. Nevertheless, it has also been reported that there is no distinction between conventional, nonbiodegradable plastics and biodegradable plastics, and they have been considered the most polluting single-use plastics [3]. Notably, biodegradable properties depend on the plastic properties including chemical structure and crystallinity and the environmental conditions, such as humidity, temperature, and other conditions [4]. Nowadays, biodegradable plastic products can be disintegrated or composted only in particular environments such as in industrial composting facilities rather than in natural environments [5]. To realize the potential environmental benefits of using bioplastics instead of conventional plastics, there is a need for the development of truly biodegradable polymers as well as biodegradability testing and standards, and factual information on the features, suitable usage, disposal, and restrictions of biodegradable plastics and their applications to be provided to relevant customers.

Among a wide range of bioplastics, polyhydroxyalkanoates (PHAs) are one of the most well-known types of biodegradable polymers with great potential to alleviate conventional plastic pollution [6]. PHAs exhibit industrial compostability, home compostability and biodegradability in open natural ecosystems. However, the production cost and mechanical properties of PHAs remain controversial. To reduce the cost of PHAs, one of our interests is to develop biocomposites of poly(3-hydroxybutyrate) (PHB) and natural fibres derived from agricultural waste [7]. Recently, biocomposites between PHB and other natural polymers, such as natural rubber, starch, wood and cellulose, have also been investigated [8–12]. As a research prototype, PHB produced from soil-isolated *Cupriavidus necator* strain A-04 was used as a major matrix material [13–15]. Regarding natural fibre resources, the agricultural sector has played an important role in the Thai economy. Pineapple is an important economic crop of Thailand and Asian countries such as the Philippines, Taiwan, and Malaysia. Currently, Thailand is one of the world’s greatest pineapple exporters. During the past five years, Thailand’s pineapple production has been claimed to dominate the world’s pineapple supply by more than 50% by exporting to other countries across the globe at approximately 2 million tons a year. It was estimated that approximately 80% of edible fruit proceeds to the industrial canning process, whereas the remaining 20% is sold for domestic consumption. The total agriculture area of 100,000 ha covers over thirteen provinces. Production was once a free-trade business, but it has since evolved into a contract business [16]. In Thailand, the Thai pineapple cultivars can be divided into three morphological groups, smooth cayenne, queen and Spanish, and subdivided into fourteen pineapple varieties [17]. The pineapple used in this study was the Batavia pineapple breed grown in Amphoe Sriracha, Chonburi Province, Thailand, which was registered for geographical indication on August 15, 2005, as Sriracha pineapple. Sriracha pineapple is a major agricultural product of Thailand that is used to produce canned pineapple, preserved pineapple, pineapple juice, etc. In addition to fruits, pineapple fields yield large amounts of leaves. Pineapple leaves are considered a rich source of cellulose obtained as agricultural waste from harvesting [18]. Due to the high cellulose content of these biological wastes, the delay of pineapple leaf decomposition in fields, which takes more than two years, results in high precultivation costs. Currently, many strategies have been adopted to target the zero-waste concept by waste minimization, reusability, or conversion to high-value-added products. Process design based on the zero-waste concept has given new life to industries that no longer generate waste [19]. The zero-waste method combines waste from one procedure with raw materials from another. Based on this concept, pineapple leaves are considered a rich source of cellulose that can serve as a renewable source of raw material for the preparation of biocomposites. Furthermore, the possibility of biomass valorization by recycling plant biomass to create natural fibres from agricultural waste resources is presented in this study via the use of pineapple leaves. In the circular bioeconomy, biomass value creation necessitates interdisciplinary techniques. Modern biorefineries and bioenergy play a vital role in biomass valorization for the production of various value-added biochemicals and biofuels in the direction of a future that is climate neutral [20]. Unfortunately, the chemical structure of PHB is hydrophobic, having the highest solubility in chloroform, and the structure of natural cellulose is insoluble in most typical organic solvents due to inter- and intramolecular hydrogen bonding [21]. The modification of cellulose by substitution of the hydroxyl group is required to enhance the solubility of cellulose in organic solvents, especially chloroform. As a result, cellulose derivatives can be processed by dissolution, melting and forming in a wide range of applications [22]. Therefore, the objective of this study was to prepare biocomposite films combining PHB produced by *C*. *necator* stain A-04 (PHB_A-04_) and long-chain fatty acid esterified microcrystalline cellulose derived from pineapple leaves as fillers. All biocomposites, including pure PHB_A-04_ and esterified pineapple leaf microcrystalline cellulose laurate (esterified PALF-MCC laurate), were subjected to FTIR analysis, thermal analysis, film surface morphology analysis, mechanical testing and soil burial testing to ensure that the newly developed biocomposites still possess true biodegradability. The advantage of biomass valorization through the utilization of waste natural fibres was demonstrated as the small amount of esterified PALF-MCC laurate in PHB matrix would significantly reduce the cost in a modest rate.

## Materials and methods

### Materials

The biodegradable polymer matrices used in this study were PHB biosynthesized from fructose by *Cupriavidus necator* strain A-04 according to a previous report [13, 23]. Batch cultivation was performed in a 10-L bioreactor (MDL-10L, B.E. Marubishi Co., Ltd., Tokyo, Japan). Culture samples were periodically harvested for analysis of the dry cell weight (DCW), PHB, carbon and nitrogen concentrations. The harvested cells were dried, placed in filter paper (Whatman 1002–042, Sigma–Aldrich Corp., St. Louis, MO, USA) and then refluxed in hot chloroform in a Soxhlet apparatus to extract PHB_A-04_ from the dried cells. The PHB_A-04_ was precipitated from a chloroform solution using three volumes of *n*-hexane. The precipitation step was repeated three times [24, 25].

As a binder and filler, the agro-industrial residue used in this study was pineapple leaves (*Ananas comosus* L. Merr.) obtained from Siam Food Products Public Company Limited (Rai Nong Takhian at Tambol Khlong Kaeo, Amphoe Banbung, Chonburi, Thailand). The pineapple leaves were washed repeatedly with distilled water to remove all the dirt, immersed in 5% sodium hypochlorite solution for 1 h, cut into approximately 2 cm × 2 cm pieces, dried in a hot-air oven (UN55, Memmert Co., Ltd., Schwabach, Germany) at 65°C for 24 h, milled using a laboratory blender (45,000 rpm, 1800-W, Healthy mix GP 3.5, Taiwan) and then sieved to fractionate the particle sizes between 0.420 and 0.250 mm (− 40/+ 60 mesh). The chemical composition of the dried pineapple leaves was determined according to the Technical Association of Pulp and Paper Industry (TAPPI) standard methods for the following parameters: benzene extractives (TAPPI T204 cm-07); α-cellulose, β-cellulose, and γ-cellulose (TAPPI T203 om-09); holocellulose (TAPPI T9 m-54; lignin (TAPPI T222 om-15); and ash (TAPPI T-211). Dried pineapple leaves with a particle size of approximately 2 cm × 2 cm were used to extract PALF-MCC.

N,N-Dimethylacetamide (DMAc, RCI Labscan Ltd., Thailand) and anhydrous lithium chloride (LiCl, ≥ 98%, Elago Enterprises Pty Ltd., N.S.W., Australia) were used as solvents, while N,N-dimethyl 1-4-aminopyridine (DMAP, 98%, Fluka) was applied as the catalyst. The esterifying agent was lauroyl chloride (TCI Co., Ltd., Tokyo, Japan). Hydrochloric acid (Merck KGaA, Darmstadt, Germany) was used for hydrolysis, and ethanol (Merck KGaA, Darmstadt, Germany) was used as the precipitating agent.

### Analytical methods

The PHB_A-04_ in dried cells was subjected to methyl-esterification [26]. The resulting fatty acid methyl esters were quantified by gas chromatography (Model CP3800, Varian Inc., Walnut Creek, CA, USA) using a Carbowax-PEG capillary column (0.25 μm df, 0.25 mm ID, 60 m length, Varian Inc.). The internal standard was benzoic acid, and the external standard was PHB (Sigma-Aldrich Corp.). The concentration of total reducing sugars was quantified using a 3,5-dinitrosalicylic acid (DNSA) assay [27], and the NH^+4^ concentration was determined through a colorimetric assay [28]. The molecular weights (*M*_W_ of 313,684 Da, M_*N*_ of 105,390 Da and polydispersity index (PDI) of 2.9) were determined by gel permeation chromatography (GPC; Shimadzu 10A GPC system, Shimadzu Co., Ltd., Kyoto, Japan) with a 10A refractive index detector and two Shodex columns (a GPC K-806M column (8.0 mm ID × 300 mm L, Showa Denko K.K., Tokyo, Japan). Polymer was dissolved in 0.1% (w/v) chloroform and filtered through a 0.45 μm low-protein-binding Durapore® (PVDF) membrane filter (Millex®-HV, Merck Millipore Ltd., Tullagreen, Carrigtwohill Co., Cork, Ireland). The operating temperature was 40°C, and the flow rate was 0.8 mL/min. A standard curve was determined for polystyrene with low polydispersity under the same conditions for molecular weights of 1.26×10^3^, 3.39×10^3^, 1.30×10^4^, 5.22×10^4^, 2.19×10^5^, 7.29×10^5^, 2.33×10^6^ and 7.45×10^6^. The weight-average molecular weight (M_W_) and the number-average molecular weight (M_N_) were determined by gel permeation chromatography (GPC), and the polydispersity index (PDI) was calculated as the ratio $\frac{M_{W}}{M_{N}}$.

### Preparation of pineapple leaf fibre microcrystalline cellulose

PALF-MCC was prepared according to the method of Suchaiya and Aht-Ong with some modifications [29]. First, 250 g of dried pineapple leaf, cut to a size of 2 cm × 2 cm, was delignified with 0.5 M sodium hydroxide solution for 2 h and then heated with continuous stirring at 80°C for 4 h to obtain PALF. The obtained PALF was washed repeatedly with distilled water to attain neutral pH and then bleached with 10% v/v H_2_O_2_ in 0.5 M NaOH solution at 80°C for 2 h. Subsequently, the bleached PALF was washed with distilled water until neutral pH was attained and hydrolysed with 2 M HCl under vigorous stirring at 80°C for 4 h. The suspension was washed with distilled water until the pH value was neutral, followed by filtration to obtain PALF-MCC. Finally, the PALF-MCC was dried in an oven at 60°C overnight. The final PALF-MCC was ground using a blender to decrease its agglomeration for 4 min and dried in a desiccator until use.

### Preparation of pineapple leaf fibre microcrystalline cellulose ester as a filler

PALF-MCC was subjected to cellulose esterification by a homogeneous acylation reaction following the method described by Suchaiya and Aht-Ong with some modifications [29]. Briefly, 2 grams of dried PALF-MCC was resuspended in 50 mL of the cosolvent of LiCl/DMAc solution at 80°C for 60 min. Next, 0.67 g of DMAP catalyst and modifying agent (lauroyl chloride) were added into the solution. The solution was heated to 60°C for 12 h and then precipitated in ethanol. The PALF-MCC ester powder was obtained after filtration and drying in an oven at 60°C for 12 h.

### Characterization of esterified PALF-MCC laurate

#### Determination of the percentage of weight increase

The percentage of weight increase (% *WI*) was calculated as

$$\%\;WI=\frac{W_{f}-W_{i}}{W_{i}}\times 100$$ (1)

where *W_i_* is the weight of the dried initial cellulose sample (g) and *W_f_* is the weight of the modified cellulose sample (g).

### Test of solvents for esterified PALF-MCC laurate dissolution

The solubility test was used to examine the polarity tendency of unesterified PALF-MCC compared with esterified PALF-MCC laurate. For this purpose, 25 mg samples were tested with 3 mL aliquots of various solvents (acetone, chloroform, ethanol, tetrahydrofuran (THF) and toluene) in screw-threaded, borosilicate-glass test tubes with a polytetrafluoroethylene (PTFE)-faced rubber line cap (PYREX®, Tewksbury, MA, USA). The sample was dissolved by heating in a water bath at 100°C for 4 h. Pictures were taken to illustrate the solubility of PALF-MCC before and after modification.

### Morphology study of PALF-MCC by scanning electron microscopy before and after modification

The morphology of PALF-MCC before and after modification was observed with a scanning electron microscope (SEM, JSM-6610LV, JEOL Co. Ltd., Tokyo, Japan) at an accelerating voltage of 15.0 kV. Before testing, the samples were dried in a hot air oven at 105°C for 12 h and mounted on a stub with carbon tape. Then, the samples were sputter-coated with a thin layer of gold prior to the experiment to protect the charging of the surface under an electron beam.

### Fourier transformed infrared spectroscopy (FT-IR)

The functional groups and chemical structure of PALF-MCC before and after modification were examined using Fourier transform infrared (FTIR) spectroscopy (Nicolet NEXUS 670, Thermo Nicolet, Thermo Scientific Co., Madison, WI, USA). The samples were diluted with KBr (1:100 by weight) and scanned at a resolution of 2 cm^-1^ with 64 scans in the range of 4000–500 cm^-1^.

### Preparation of PHB_A-04_/PALF-MCC laurate biocomposite films

The PHB_A-04_/PALF-MCC laurate biocomposite films containing 0, 5, 10, 15, 20 and 100% PALF-MCC laurate by weight) were prepared according to the ASTM: D882-91 protocol. The biocomposite films were obtained from conventional solvent-casting techniques using a Pyrex glass tray (Pyrex, Corning Incorporated, NY, USA) as the casting surface [30]. The thickness of the thin films was controlled at a fixed concentration of the polymer in chloroform (1% w/v) and the volume of the polymer solution. The thickness of the thin films was 0.05 mm, as measured with a calliper (Model 500–175: CD-12C, Mitutoyo Corporation, Kawasaki-shi, Kanagawa, Japan). At least five thin film samples were cut (50 × 150 mm) and kept in a desiccator at ambient temperature for 1 month to reach crystallization equilibrium before the analyses.

### Characterization of biocomposite films

#### Morphological analysis

The morphology of the biocomposite films was observed with an SEM (JSM-6610LV, JEOL Co. Ltd., Tokyo, Japan) as mentioned above.

### Thermal analysis by differential scanning calorimetry (DSC) and thermogravimetric analysis (TGA)

Samples with a weight of 10 mg were subjected to thermal analysis by the DSC apparatus (DSC 204 F1 Phoenix®, NETZSCH Thermal Analysis, NETZSCH-Gerätebau GmbH, Selb, Germany). The thermal history background of the sample was removed before thermal analysis by heating the sample from ambient temperature to 180°C at 10°C/min and maintaining the sample at 180°C for 5 min before cooling to −50°C. Next, the sample was heated at 10°C/min to 180°C. The melting peak temperature (T_m_) was defined as the intersection of the tangent to the farthest point of an endothermic peak and the baseline. The glass transition temperature (T_g_) was estimated by extrapolating the midpoint of the heat capacity difference between glassy and viscous states after heating of the quenched sample.

Thermogravimetric analysis (TGA) was performed with a TGA 7 (Perkin-Elmer Inc., Waltham, MA, USA). Samples with a weight of 10 mg were heated from 30°C to 800°C at 10°C/min under nitrogen atmosphere with a flow rate of 50 mL/min.

### Analysis of the mechanical properties of PHB/esterified PALF-MCC laurate biocomposite films

The thin films prepared according to ASTM: D882-91 protocol were subjected to mechanical tests using a Universal Testing Machine (H10KM, Wuhan Huatian Electric Power Automation Co., Ltd., Wuhan, China). The crosshead speed was 10 mm/min. The obtained variable parameters were the elongation at the break point (%), the stress at maximal load (MPa) and Young’s modulus (MPa). For comparison, the thin films of PHB (Sigma-Aldrich Corp.) were also tested under the same conditions. The data represent the mean values ± standard deviations (SDs) of at least five samples.

### Soil burial degradation process

Biocomposite film degradation by soil burial method was performed in an indoor environment for 3 months according to Rizzarelli et al. and Phetwarotai et al. with some modifications [31, 32]. A plastic box containing fertilized soil (inorganic nitrogen 0.35%, inorganic phosphorus 0.18%, inorganic potassium 1.37%, soil electrical conductivity 1.2 deciSiemens/m, soil organic matte 7.71%, pH 6.69) was prepared. The carbon to nitrogen ratio was 22.03%. The biocomposite films were cut into 2 × 2 cm pieces and dried at 60°C for 24 h. The dried film was placed in a mesh bag (8 cm × 4 cm) containing 2 g of fertilized soil and buried in soil at a 30 cm depth from the surface for 3 months. The pH, temperature and moisture content were monitored throughout the test. The pH values of soil were in the range of 6.5 to 7.5, the soil moisture content was maintained in the range of 80 to 100% by watering every 24 h, and the temperature was in the range of 20 to 28°C. The samples were removed and tested for degradation every week. After removal, the film samples were washed with distilled water and dried in a vacuum oven until reaching a constant weight. The degree of degradation was evaluated by determining the changes in physical appearance, surface morphology by SEM analysis and percentage weight loss. Experiments were independently repeated 3 times.

### Observation of PHB/PALF-MCC laurate biocomposite film degradation by scanning electron microscopy

The surface morphologies of the thin film samples before and after degradation were determined with a scanning electron microscope (SEM, JSM 6480, JEOL, Tokyo, Japan) operating at 15 kV. Each sample was washed with distilled water and dried in a vacuum at 40°C until a constant weight before testing. The surface of the biocomposite films was coated with gold prior to investigation to avoid surface charging under an electron beam.

### Determination of weight loss

The weight loss of the biocomposite films was calculated by subtracting the weight of film samples after subjecting them to the soil burial test from the initial weight at every regular time interval (7 days). The weight loss of the film samples with time was defined as the degradation rate in the soil burial test. The percentage weight loss was calculated using the following equation:

$$\mathrm{Weight}\;\mathrm{loss}\;(\%)=\frac{W_{i}-W_{f}}{W_{i}}\times 100$$ (2)

where *W_i_* is the initial weight of the sample before testing (g) and *W_f_* is the final weight of the sample after testing (g). The degradation data revealed very fast degradation by the soil burial method used. The linear section of the curve of the biocomposites is considered as full degradation being accomplished. These data were calculated by applying an integrated kinetic equation of the first order [33, 34]

$$\ln\frac{W_{f}}{W_{i}}=kt$$ (3)

where the time is expressed in days and *k*, the kinetic constant of the first order, is expressed in days^-1^. The half-life of the biocomposite, T12, was calculated with the following equation [33, 34]

t12=ln(2)k (4)

### Data analysis

All the data presented in this research are the results obtained from three independent experiments and are calculated as the mean values ± standard deviations (SDs). Analysis of variance (one-way ANOVA) followed by Duncan’s test for testing differences among means was conducted using SPSS version 22 (IBM Corp., Armonk, NY, USA). Differences were considered significant at P < 0.05.

## Results and discussion

### Compositions of agro‑industrial residues

The compositions of PAL analysed in this study were observed to be 38.9% (w/v) holocellulose [26.9% (w/v) α-cellulose, 4.96% (w/v) β-cellulose, and 7.04% (w/v) γ-cellulose] and 9.23% (w/v) lignin. The water content was 8.2% (w/v). The results are shown in Table 1 and show that the compositions of PAL in this study were similar to those obtained from PAL grown in Thailand [35–37]. The holocellulose content, however, was somewhat lower than those reported in different countries. The lignocellulose compositions of pineapple plants vary based on the plant variety, plant age, growth conditions, soil conditions, geography, location, climate, and other environmental factors, such as temperature, stress, and humidity [38].

**Table 1:** Characterization of agro-industrial residues.

Lignocellulosic biomass compositions (%)									Reference
α-cellulose	β-cellulose	γ-cellulose	holocellulose	lignin	ash	benzene extractives	moisture (%)	Remark	
26.9	5.0	7.0	38.9	9.2	-	-	8.2	Thailand	This study
-	-	-	35.8	6.1	7.2	-	-	Thailand	[36]
16.6	-	24.9	41.6	13.9	6.0	13.1	-	Thailand	[37]
35.4		16.6	52	16	5	16		Thailand	[35]
39.7		22.7	62.4	4.1				Thailand	[65]
49.2		17.8	67.1	17.4		11.2		Thailand	[66]
51.2		19.8	71.0	2.0				Thailand	[67]
			75.1	5.0	0.9		9.5	Philippines	[68]
			70–82	5–12	1.2		11.8	India	[69]
			68.5	6.0	0.9			India	[70]
87.4		4.6	91.9	3.6	0.5	2.7	11.6	India	[71]
74.3		6.4	80.7	10.4	4.7	6.7		Malaysia	[72]
73.4		7.1	80.5	10.5	2	5.5		Malaysia	[73]

% hemicellulose = (% holocellulose)—(% α-cellulose)

The chemical compositions of pineapple leaves used in this study were determined according to the Technical Association of Pulp and Paper Industry (TAPPI) standard methods.

### Characterization of PHB produced by *C*. *necator* A-04

**Fig 1:**
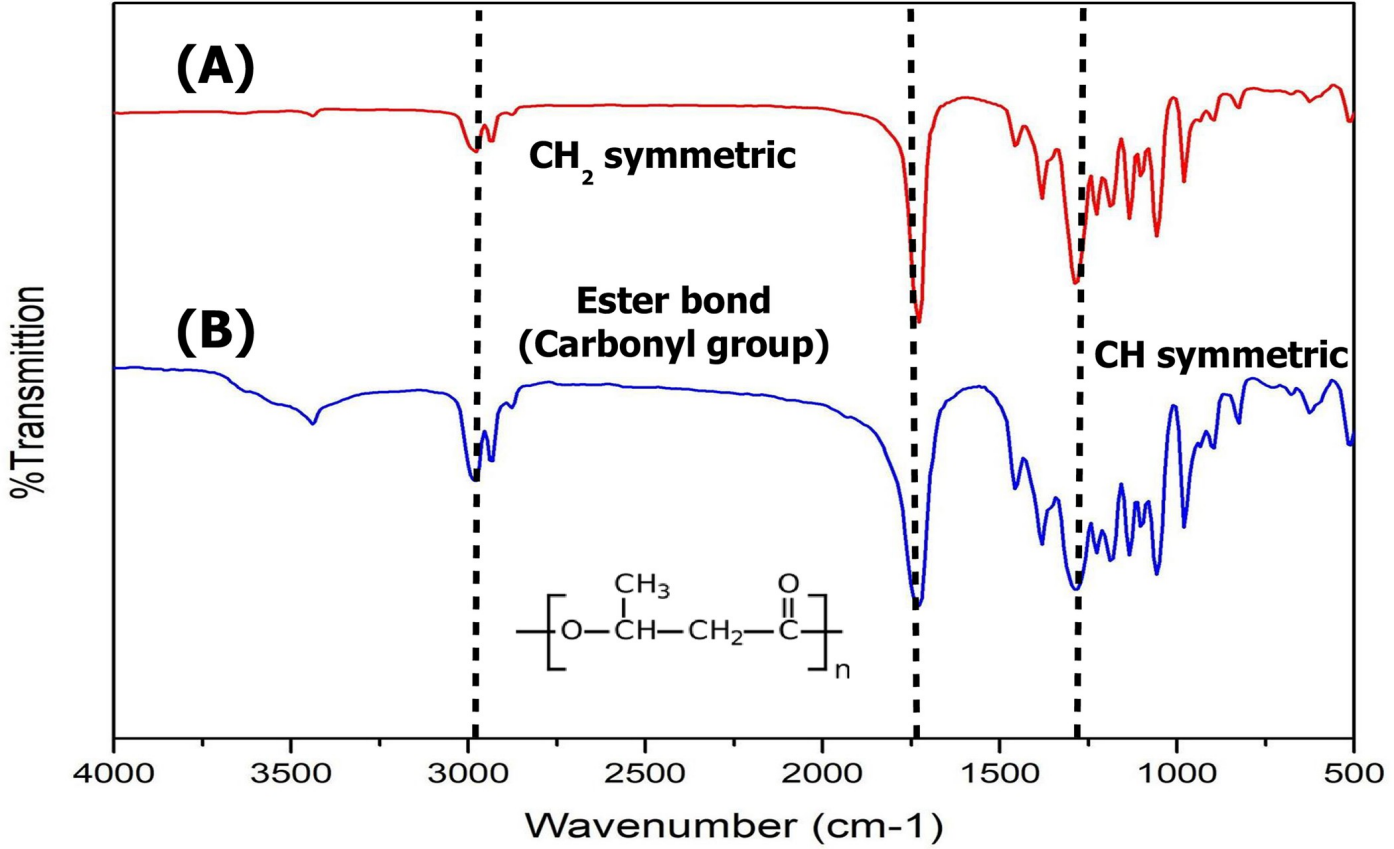
FTIR spectra of **(A)** commercial PHB (Sigma-Aldrich Corp.) and **(B)** pure PHB_A-04_ produced from *Cupriavidus necator* strain A-04 (B).

In this study, PHB produced from fructose by *C*. *necator A-*04 was chosen for the development of green composites made of PHB_A-04_ and PALF-MCC. Aerobic batch cultivation was performed in a 10 L fermenter. At 60 h, a total cell mass of 7.8 ± 0.48 g/L was obtained with a PHB_A-04_ content of 70.54 ± 5.7 wt% when fructose was used as the carbon source. Purified PHB_A-04_ was analysed by ^1^H NMR and FTIR to confirm its chemical structure in comparison with commercial PHB (Sigma-Aldrich Corp.) The FTIR spectra of the produced and commercial PHB are compared in Fig 1. FTIR chromatogram peaks appearing at a wavelength frequency of 2980 cm^-1^ indicated CH_2_ symmetry, the major functional groups were found at a wavelength frequency of 1727 cm^-1^, indicating carbonyl groups, and a wavelength frequency of 1283 cm^-1^ indicated CH symmetry [39, 40]. The ^1^H NMR chromatogram also confirmed that the produced polymer was homopolymer PHB. The weight-average molecular weight (M_W_), number-average molecular weight (M_N_) and polydispersity index (PDI) of PHB_A-04_ were 3.14×10^5^, 1.06×10^5^ and 2.9, respectively. The DSC and TGA results are presented in Table 2. The produced PHB_A-04_ showed T_g_, T_c_, T_m_ and T_d_ values similar to those of commercial PHB; thus, it can be used as a representative of commercial PHB.

**Table 2:** Thermal analysis by thermal gravimetric analysis (TGA) and differential scanning calorimetry (DSC) for commercialized PHB, produced PHB_A-04_ and produced PHB/esterified PALF-MCC at the rate of 10°C/min.

Samples	Thermal properties						
	T_m_ (°C)	T_c_ (°C)	T_g_ (°C)	T_d_ at 50% (°C)	ΔH_C_ (j/g)	ΔH_M_ (j/g)	X_C_ (%)
**Filler**							
unmodified PALF-MCC	**-**	**-**	75	330.6	**-**	**-**	**-**
esterified PALF-MCC laurate	**-**	**-**	59.3	355.9	**-**	**-**	**-**
**major matrix**							
PHB (Sigma-Aldrich)	175.4	48	3.5				
PHB_A-04_	172.8	53.4	3.6	261.0		99.70	68.29
**biocomposite** PHB _A-04_/esterified PALF-MCC laurate							
95:5	173.1	67.9	3.0	271.8		81.96	59.09
90:10	172.4	63.1	2.4	279.1		77.74	62.37
85:15	172.9	65.3	3.2	276.9		72.55	58.46
80:20	172.2	63.8	3.4	270.6		70.69	60.52

nd = not detected

### Comparison of FTIR spectrum, solubility, morphology and thermal properties between PALF-MCC and PALF-MCC laurate

#### Chemical structure by FTIR

**Fig 2:**
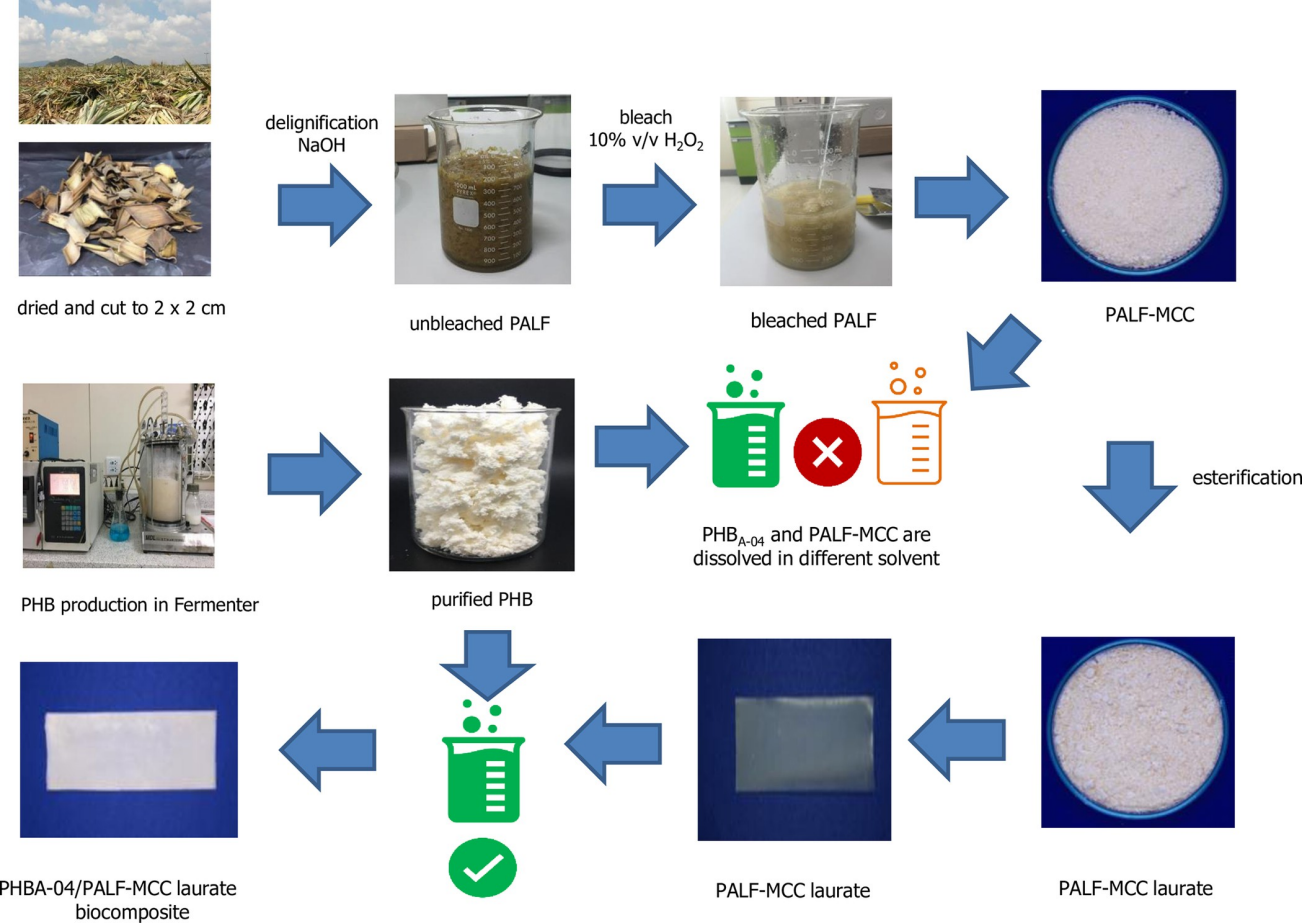
Schematic diagram for the preparation of esterified PALF-MCC laurate to depict the method for preparing fibres to be compatible with solvent casting PHB_A-04_.

**Fig 3:**
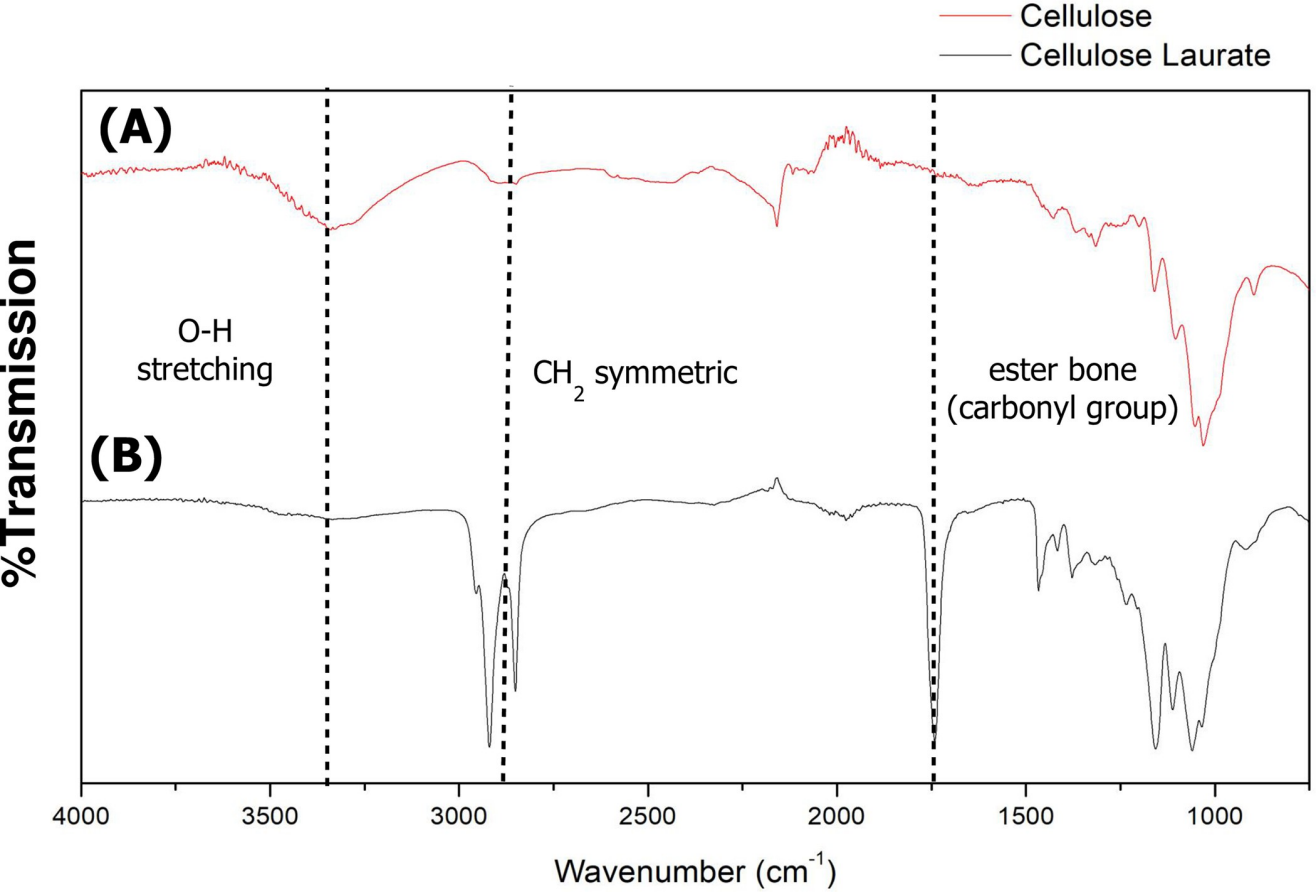
FTIR spectra of PALF-MCC and its esterification with fatty acid chloride. **(A)** FTIR spectra of native PALF-MCC and **(B)** esterified PALF-MCC laurate.

The esterification reaction of PALF-MCC was carried out under a DMAc/LiCl solvent system using DMAP as a catalyst. The schematic diagram for the preparation steps of the esterified PALF-MCC laurate is shown in Fig 2. FTIR was used to confirm the functional groups and chemical structure of the esterified PALF-MCC laurate. The FTIR spectra of PALF-MCC before and after esterification were compared. The effect of laurate ester on the chemical structure of PALF-MCC is presented in Fig 3. The spectrum of native PALF-MCC (Fig 3A) exhibited a strong intense peak centred at 3340 cm^−1^, which is denoted as the O–H stretching vibration. The IR spectra of the esterified PALF-MCC laurate differed from that of PALF-MCC by several bands. Since the ester carbonyl group (C = O) was found at 1741 cm^-1^, these spectra demonstrate that esterification proceeded. The methyl and methylene C–H stretching associated with acyl substitution is responsible for the significant intense peak at approximately 2800–2900 cm^-1^ in the spectra, while the intense peak at approximately 3340 cm^-1^ is attributable to that the cellulose O–H vibration dropped. In addition, these esterified PALF-MCC laurate spectra showed an intense ester carbonyl peak at 1741 cm^−1^ [22].

### Solubility properties

**Fig 4:**
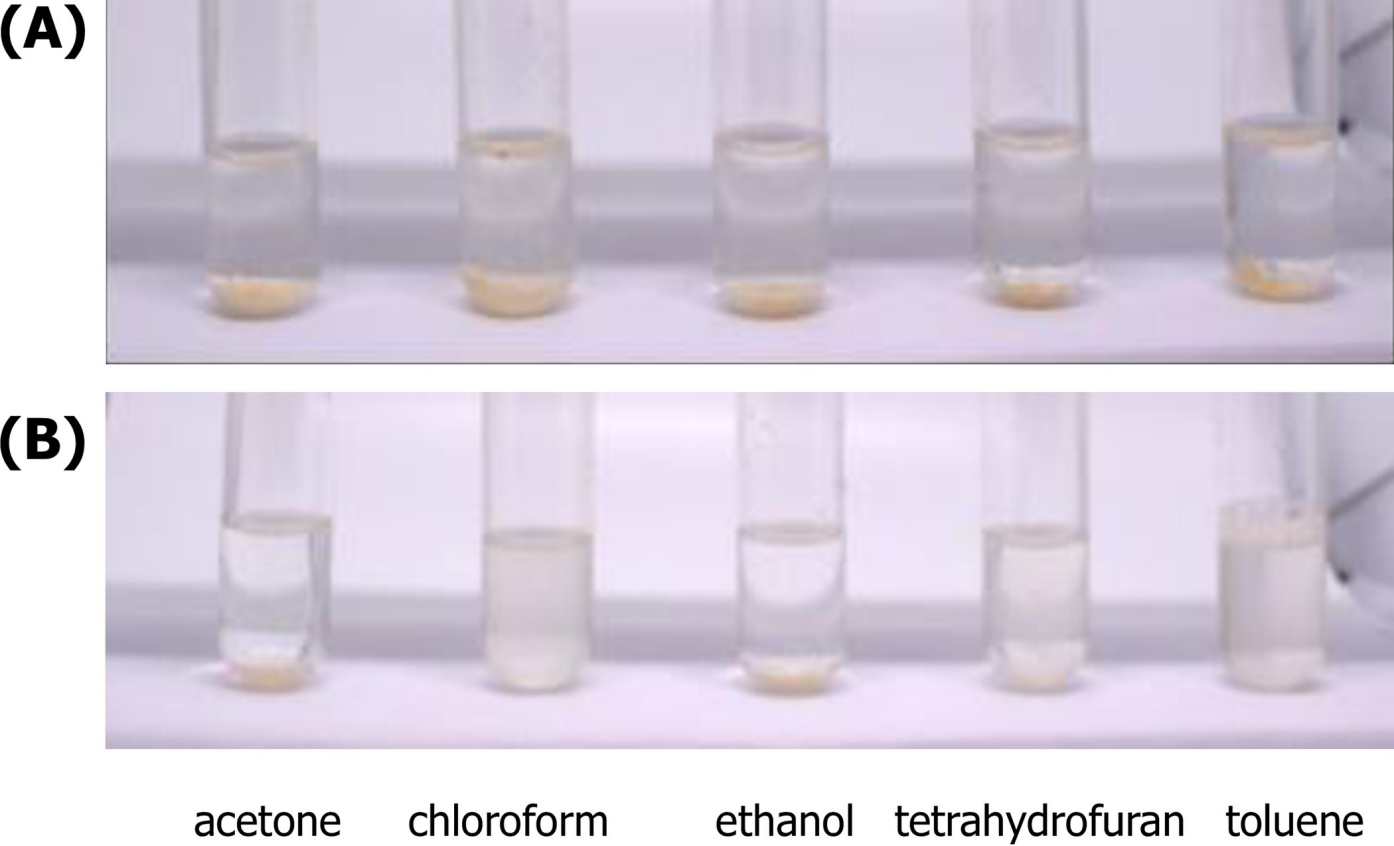
Comparison of solubility tests in acetone, chloroform, ethanol, tetrahydrofuran and toluene for (A) native PALF-MCC and (B) esterified PALF-MCC laurate.

The solubility of the unesterified PALF-MCC and the esterified PALF-MCC laurate in organic solvents in various forms was studied at a concentration of x% (w/v). Fig 4 shows the solubility test of the unesterified PALF-MCC (Fig 4A) and the esterified PALF-MCC laurate (Fig 4B) in various solvents. It was observed that PALF-MCC was insoluble in both water and organic solvents. Crystals form when the hydroxyl group of the cellulose chain absorbs moisture and forms intra- and intermolecular hydrogen bonds with neighbouring cellulose chains. Consequently, native cellulose is insoluble in both aqueous and organic solvents. After esterification, when hydrophobic acyl groups are added into the molecular structure of cellulose, highly structured hydrogen-bonding of crystal structures is destroyed, affecting the solubility. As shown in Fig 4, the esterified PALF-MCC laurate was completely dissolved in nonpolar solvents. The highest solubility was obtained in chloroform and tetrahydrofuran, followed by moderate dissolution in toluene. Due to the increase in hydrophobicity or decrease in polarity related to the acyl substituent of the esterified PALF-MCC laurate, the esterified PALF-MCC laurate could not be dissolved in polar solvents (ethanol and acetone), which also increased the solubility in nonpolar solvents such as chloroform [22]. In addition, the %WI of the esterified PALF-MCC laurate was increased to 162%, similar to previous reports [22]. The increase in %WI for the esterified PALF-MCC laurate was due to the grafting of acyl substituents. Therefore, lauroyl chloride with a molecular weight of 218.77 g/mol, which was used as an esterifying agent, had a higher %WI of the esterified product than unesterified PALF-MCC.

### Morphology

**Fig 5:**
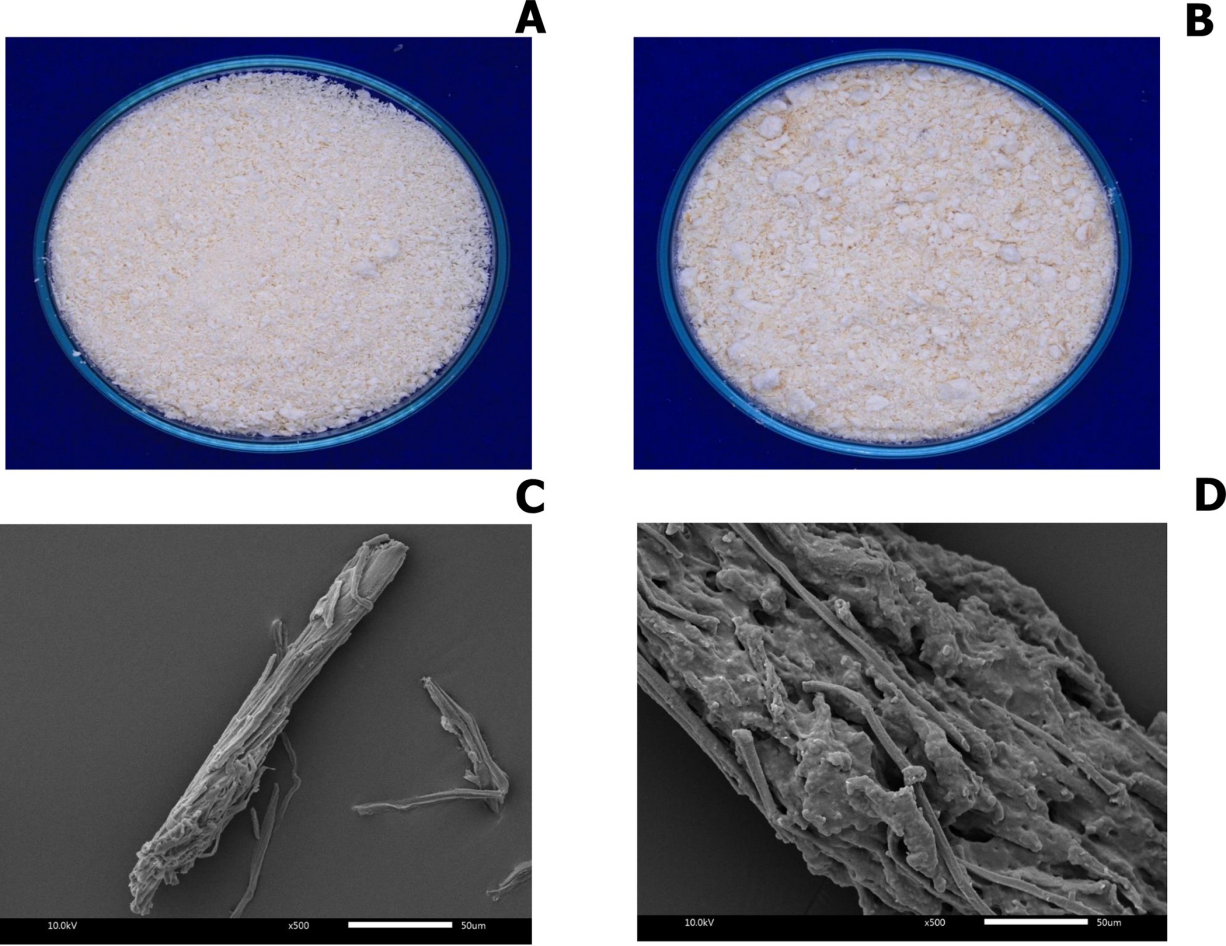
Appearance of (A) native PALF-MCC powder, (B) esterified PALF-MCC laurate powder, SEM micrographs of **(C)** native PALF-MCC and **(D)** esterified PALF-MCC laurate.

After acid hydrolysis of pineapple leaf fibre waste, on the macroscopic scale, the fibres became off-white powder (Fig 5A and 5B). The SEM analysis exhibited a different structure, as illustrated in Fig 5C and 5D. The morphology of native PALF-MCC fibres showed short and smooth surface (Fig 5C). Obviously, the esterified PALF-MCC laurate possessed large and rough surface (Fig 5D). The acyl substitution of lauroyl chloride as an esterifying agent resulted in the aggregation of acyl substituent groups on the PALF-MCC fibres according to these results. The particle size of PALF-MCC prepared in this study was approximately 15 μm in diameter, whereas the particle size of the esterified PALF-MCC laurate was approximately 132 μm. Therefore, the SEM micrographs confirmed the incorporation of lauroyl chloride in the PALF-MCC matrix.

### Thermal properties

**Fig 6:**
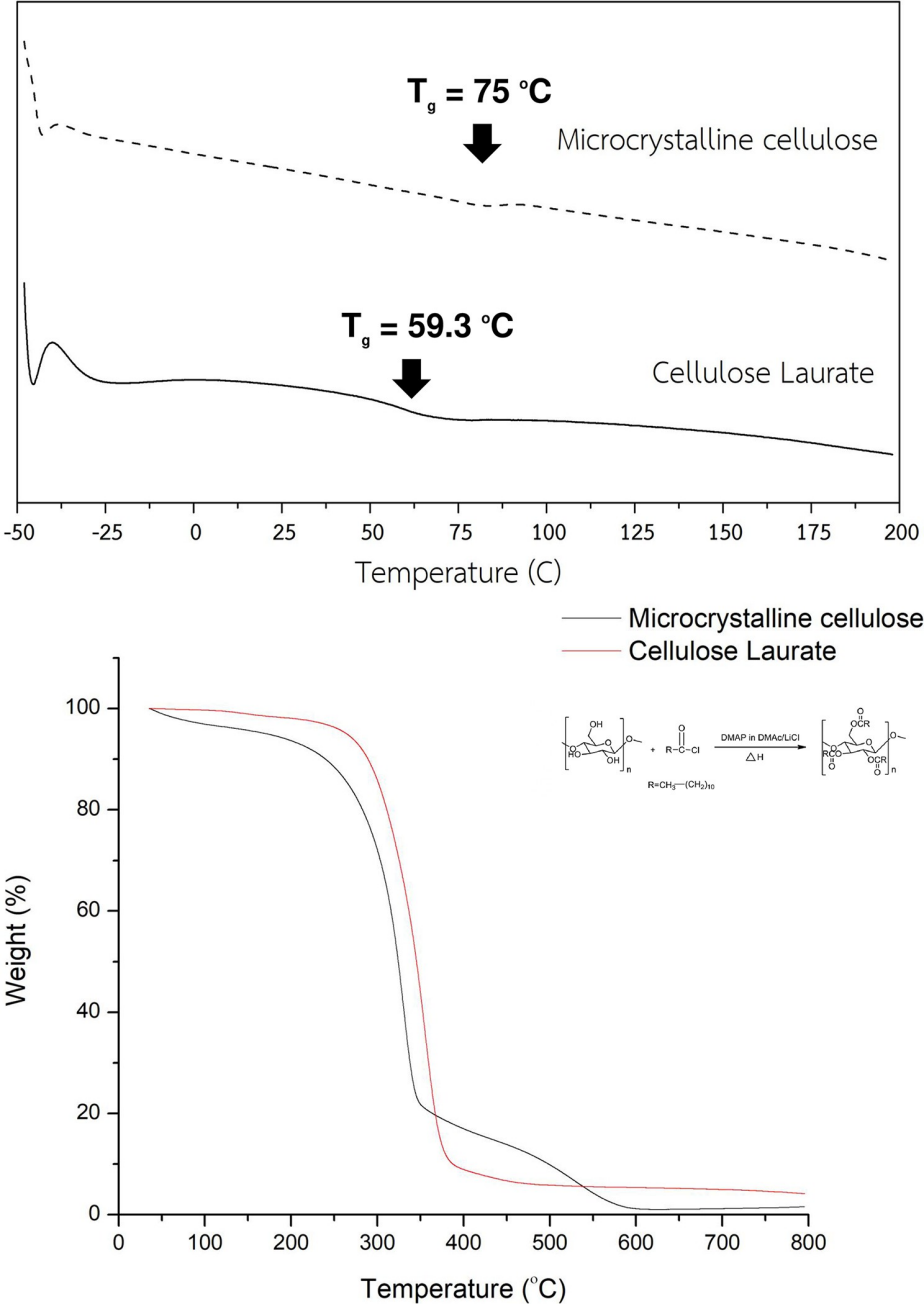
DSC thermograms **(A)** of native PALF-MCC and esterified PALF-MCC laurate and TGA thermograms **(B)** of native PALF-MCC and esterified PALF-MCC laurate.

The effect of a C_12_ lauroyl chloride as an esterifying agent on the thermal stability of the esterified PALF-MCC laurate is shown in Fig 6A. The TGA curve of native PALF-MCC exhibits three stages of decomposition. The absorbed water content in the cellulose structure is the first stage, which occurs at 90–100°C and results in a total weight loss of approximately 10%. The second stage, which occurs at a higher temperature of 300–350 ^๐^C, is caused by the decomposition of cellulose, which results in a total weight loss of 70.25%. The third stage, at 400–650°C, is from the decomposition of residual carbon and hemicellulose, accounting for 15.35% of the total weight loss. After esterification, the esterified PALF-MCC laurate exhibits two decomposition stages. The first one at approximately 150°C is the decomposition of esterified aliphatic chains from substitution reactions between the hydroxyl groups present at the surface of cellulose and long-chain fatty acid chlorides in the cellulose structure. The second stage, occurring at a higher temperature of approximately 250–380°C, is due to the decomposition of cellulose. The esterified PALF-MCC laurate does not present a decomposition stage of absorbed water because it contains hydrophobic esterified laurate, confirming that the esterified PALF-MCC laurate was more hydrophobic than the unesterified PALF-MCC. TGA curves of the esterified PALF-MCC laurate revealed a significant single weight loss stage due to cellulose degradation. There was no indication of absorbed water decomposition, indicating that esterified PALF-MCC laurate was more hydrophobic than native PALF-MCC. The thermal degradation of cellulose ester was affected by addition of C_12_ lauroyl chloride, and it decreased from 355.85°C to 330.57°C. As shown in Fig 6B, the T_d_ onset of PALF-MCC was approximately 355.85°C, whereas the PALF-MCC laurate revealed a T_d_ onset of approximately 330.57°C. The decrease in thermal stability of cellulose ester due to the decrease in crystallinity caused by substituting C_12_ acyl groups caused the T_d_ onset to shift toward lower temperatures. The thermal stability of cellulose ester is worse than that of original cellulose according to our findings [22, 41, 42].

### Effect of the content of esterified PALF-MCC laurate on the mechanical properties of PHB_A-04_/ esterified PALF-MCC laurate biocomposite films

**Fig 7:**
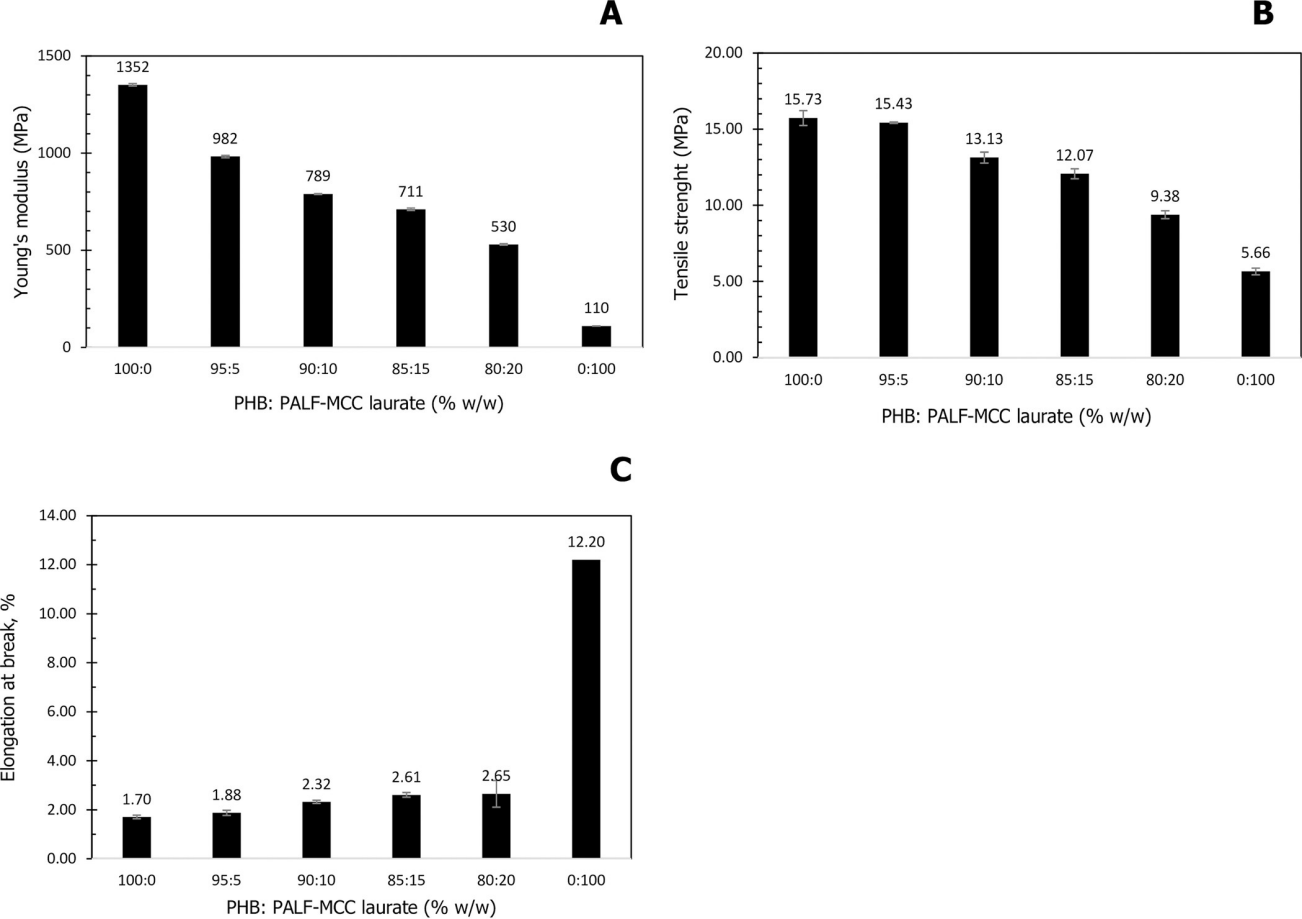
Effect of esterified PALF-MCC laurate addition on mechanical properties: (A) Young’s modulus, (B) tensile strength and (C) % elongation at break of PHB/esterified PALF-MCC laurate films.

The effects of the esterified PALF-MCC laurate content on the mechanical properties, such as tensile strength, Young’s modulus, and elongation at break, of PHB_A-04_/esterified PALF-MCC laurate biocomposite films were studied and are shown in Fig 7A–7C. The content of esterified PALF-MCC laurate as a filler was tested at 0, 5, 10, 15, 20 to 100% in biocomposite films. Young’s modulus of PHB_A-04_ was 1352 ± 6.3 MPa, the tensile strength was 15.73 ± 0.5 MPa, and the elongation at break was 1.70 ± 0.05%, whereas Young’s modulus of esterified PALF-MCC laurate was 110 ± 0.3 MPa, the tensile strength was 5.66 ± 0.2 MPa, and the elongation at break was 12.20 ± 0.5%. Fig 7A shows the Young’s modulus of the composites as a function of the esterified PALF-MCC laurate content. The Young’s modulus of neat PHB_A-04_ decreased with esterified PALF-MCC laurate addition from 1352 ± 6.3 to 530 ± 4.0 MPa due to the low modulus of esterified PALF-MCC laurate (110 ± 0.3 MPa). Fig 7B shows the tensile strength values of PHB_A-04_/esterified PALF-MCC laurate composites. A maximum tensile strength of 15.73 MPa was obtained with PHB_A-04_. Adding esterified PALF-MCC laurate 5% (w/w) did not yield a decline in the tensile strength of PHB/esterified PALF-MCC laurate composites. However, the tensile strength decreased from 13.13 ± 0.4 MPa to 9.38 ± 0.3 MPA as the esterified PALF-MCC laurate content increased from 10 to 20% (w/w). The applications of lignocellulosic fibres as fillers have been reported, but there are also some hindrances such as incompatibility between lignocellulosic fibres and other biopolymers due to differences in their affinity for water [43, 44]. However, the hydrophilicity and strong crosslinking of unmodified lignocellulosic fibres prevent the compatibility with hydrophobic matrices of biopolymers, leading to poor interfacial adhesion and mechanical properties [45, 46]. In addition, unmodified lignocellulosic fibres possess low stability when exposed to moisture [45]. Therefore, surface modification techniques are generally adopted to enhance the performance of lignocellulosic fibres and to promote better adhesion between the natural fibre and biopolymeric matrix [43]. The objectives of surface modification of the fibre via chemical reactions are to decrease the moisture content, decrease the dimensional instability, and produce more reaction sites to react with hydrophobic matrix. The goal of this study was to use a simple method involving less energy and processing steps. In this study, esterification was employed as an accepted technique for surface modification due to the high amount of free hydroxyl groups available at the surface of lignocellulosic fibres [47]. The compatibility between PHB_A-04_ and esterified PALF-MCC laurate can be investigated based on morphological observation using SEM analysis, as shown below.

### Effect of the content of PALF-MCC laurate as filler in PHB_A-04_/esterified PALF-MCC laurate biocomposite films on the fracture surface

**Fig 8:**
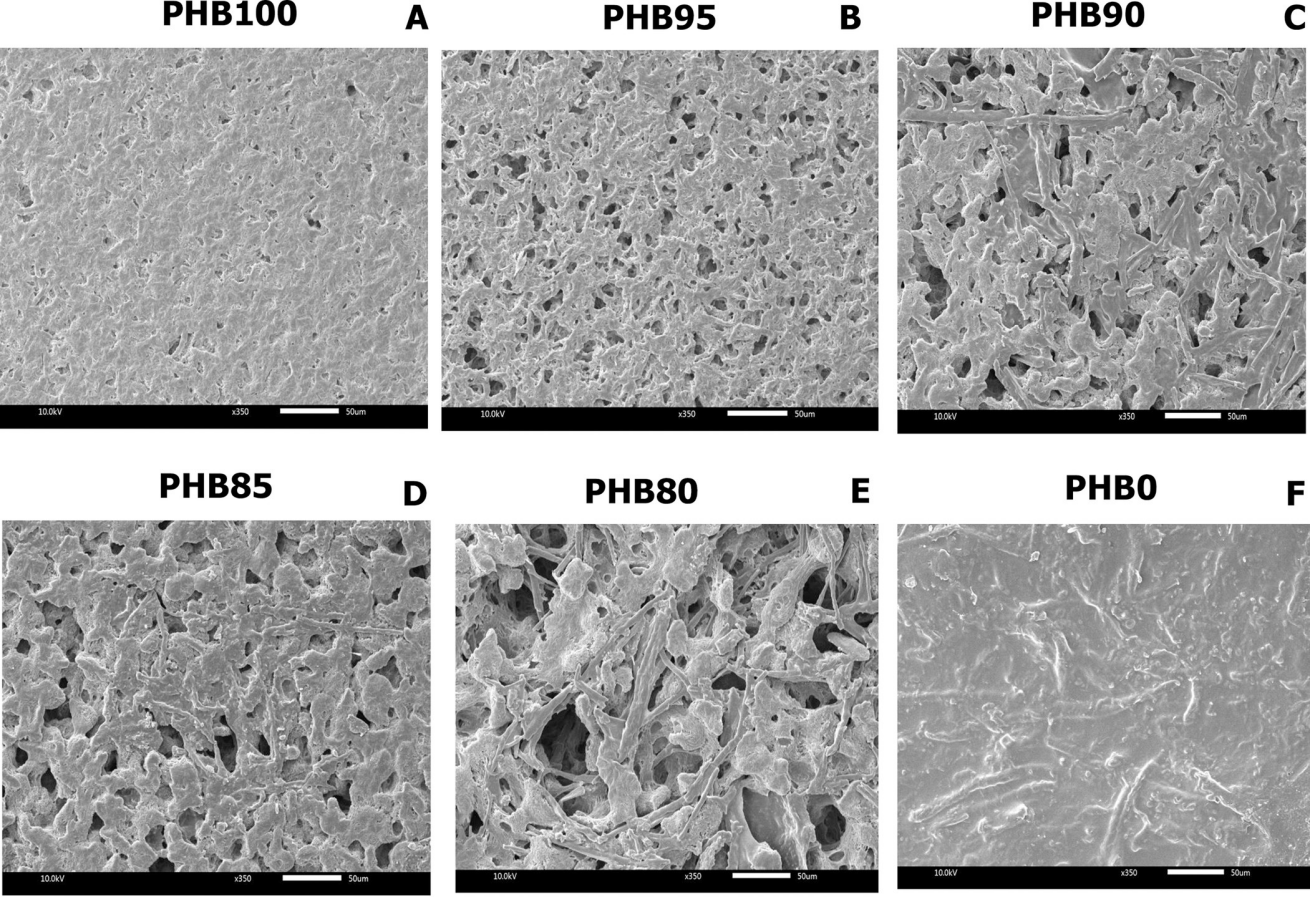
SEM micrographs of the surface morphology of PHB/esterified PALF-MCC laurate biocomposite films.

To evaluate the compatibility of esterified PALF-MCC laurate in the PHB_A-04_ matrix, the dispersal behaviour was investigated by morphological observations using SEM (Fig 8). With the addition of 5% esterified PALF-MCC laurate, Fig 8B shows the compatibility of esterified PALF-MCC laurate in the PHB_A-04_ matrix, and thus, the tensile property was similar to that of the PHB_A-04_ matrix, which can probably be attributed to the filler effect by the esterified PALF-MCC laurate that maintain a uniform stress distribution from the biopolymer matrix to the dispersed matrix phase. However, the surface morphology at a higher content of esterified PALF-MCC laurate showed an uneven distribution of the PHB_A-04_ matrix and esterified PALF-MCC laurate. Fig 8C and 8D demonstrate that the surface morphology of the sample with the amount of esterified PALF-MCC laurate higher than 5% in composites was less consistent, as indicated by the presence of more voids and agglomeration on the fracture surface due to the high volume of esterified PALF-MCC laurate used, which has a detrimental effect on the structural integrity [48, 49]. Moreover, the reduction of tensile strength might be associated with a higher esterified PALF-MCC laurate content, which was incompatible with dispersion in the matrix with an increasing esterified PALF-MCC laurate content. As a result, excessive esterified PALF-MCC laurate content led to insufficient PHB matrix–esterified PALF-MCC laurate interactions and inadequate interfacial adhesion [50, 51]. This finding was proven by the occurrence of some voids on the matrix surface and agglomerated matrix, as seen in the SEM micrograph of the fracture surface. A similar phenomenon was also observed in the development of thermoplastic starch [52].

**Fig 9:**
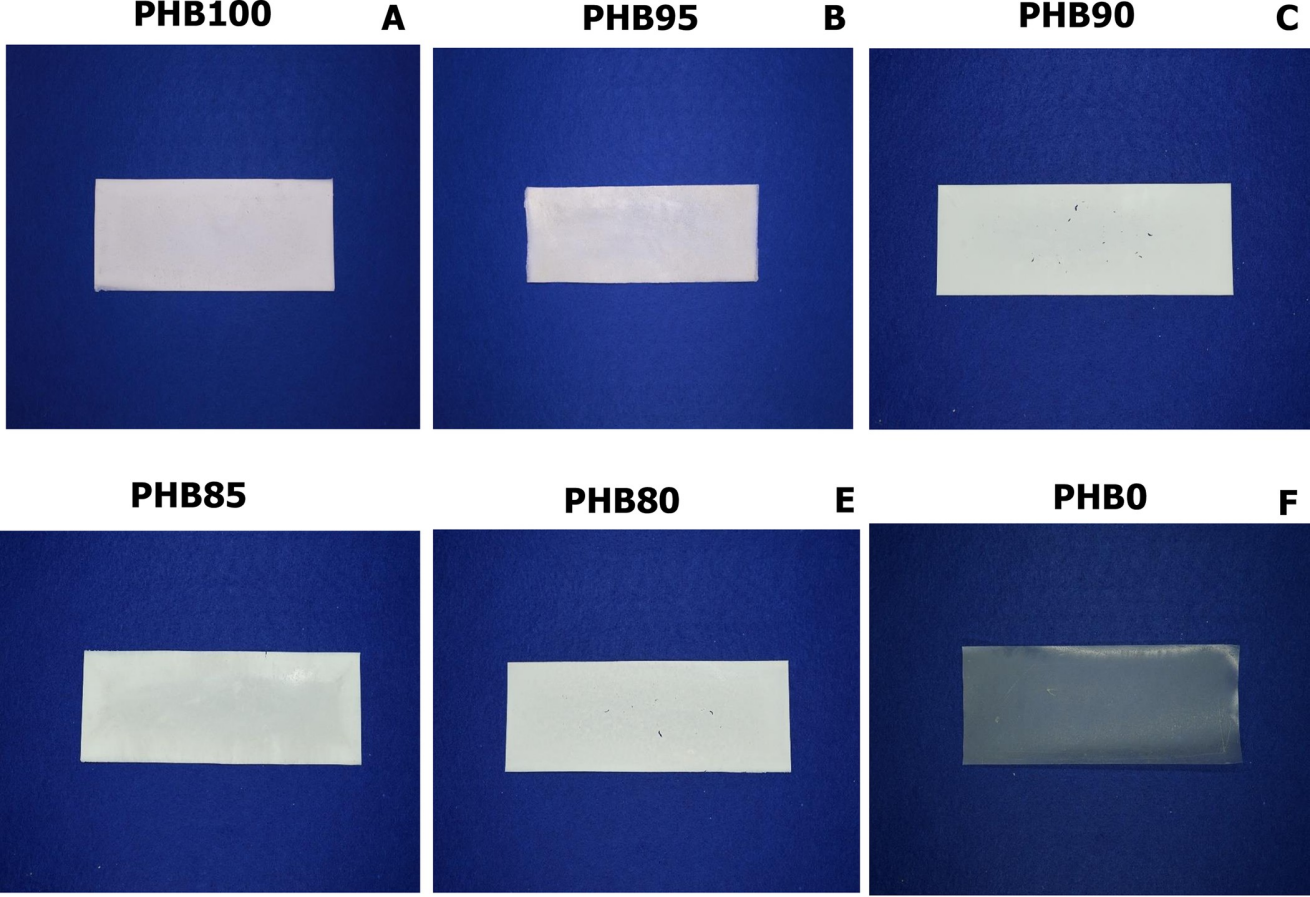
Appearance of PHB/esterified PALF-MCC laurate biocomposite films.

From our previous study, 100% PHB showed brittleness and stiffness properties such as high strength and low elongation at break [15, 25]. In this study, the presence of 5% (w/w) esterified PALF-MCC laurate as a filler offered compatibility between PHB_A-04_ and esterified PALF-MCC laurate, which resulted in maintaining a high enough value for tensile strength and elastic modulus as Yong’s modulus decreases with the addition of esterified PALF MCC laurate. Thus, 5% (w/w) esterified PALF-MCC laurate was the optimal content. In addition, lightly increasing elongation will eventually increase flexibility. As shown, the 100% PHB_A-04_, 95:5 w/w PHB_A-04_/esterified PALF-MCC laurate and 100% composite had smoother surfaces than composites containing more than 5% (w/w) esterified PALF-MCC laurate, which may be the maximum content to achieve optimal compatibility and interfacial adhesion between PHB_A-04_ and esterified PALF-MCC laurate. The appearance of film sheets made of PHB_A-04_ and esterified PALF-MCC laurate is shown in Fig 9.

### Effect of the content of esterified PALF-MCC laurate as filler in PHB_A-04_/esterified PALF-MCC laurate biocomposite films on the thermal properties

**Fig 10:**
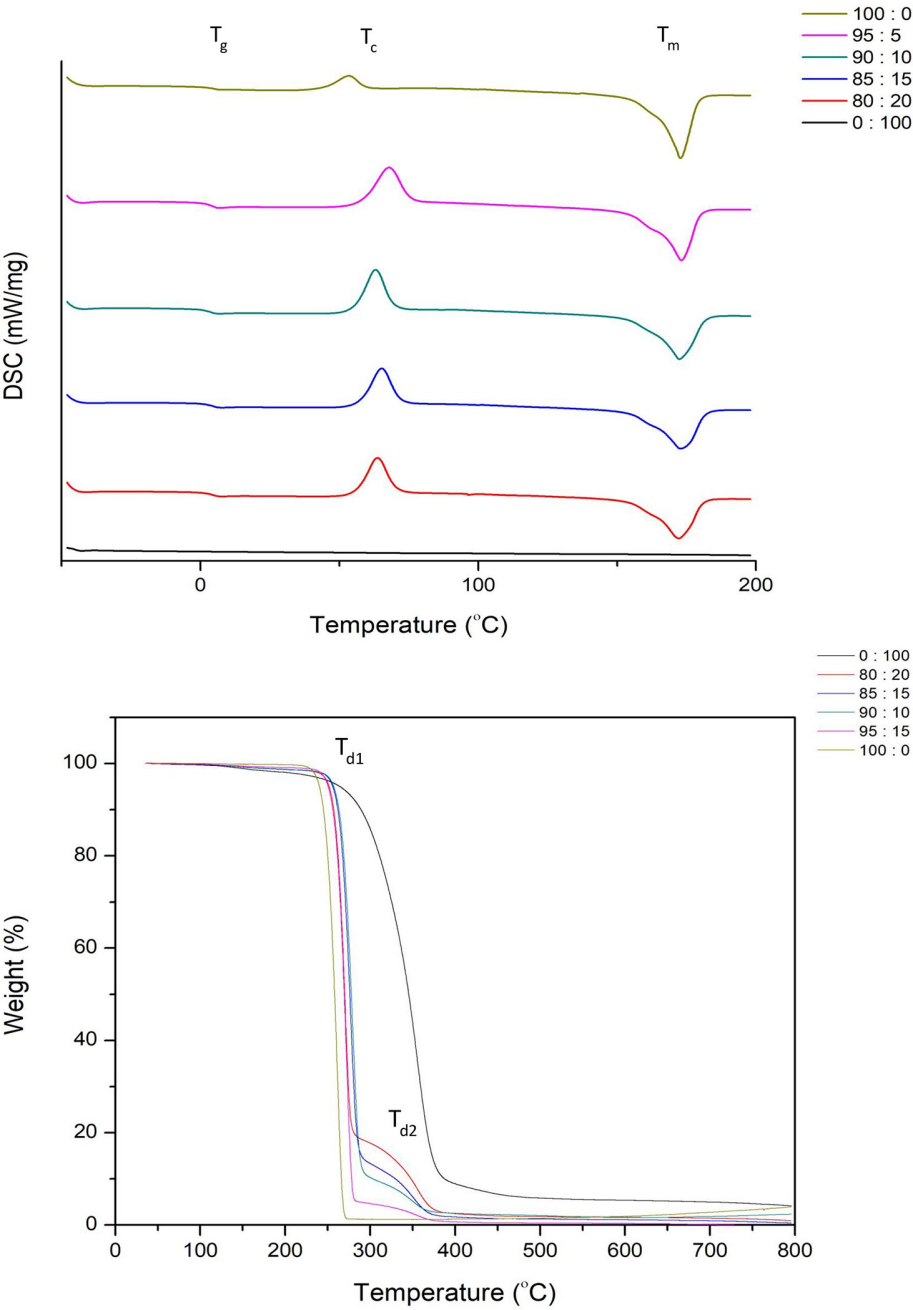
DSC thermograms **(A)** of PHB/esterified PALF-MCC laurate biocomposite films and TGA thermograms **(B)** of PHB/esterified PALF-MCC laurate biocomposite films.

The DSC and TGA thermograms of 0, 5, 10, 15, 20 and 100% (w/w) esterified PALF-MCC laurate content in PHB_A-04_/ esterified PALF-MCC laurate composites are illustrated in Fig 10A and 10B, respectively. The T_d_ of neat PHB_A-04_ appeared at approximately 261°C, whereas 100% (w/w) esterified PALF-MCC composites had a higher T_d_ than neat PHB_A-04_ at 355.9°C due to the higher thermal stability or T_D_ (at 50%) of PALF-MCC. The T_d_ of PHB_A-04_/ esterified PALF-MCC laurate composites was shifted toward a higher temperature than that without cellulose ester because the presence of ester groups grafted onto the cellulose structure increased the inter- and intramolecular hydrogen bonds and crystallinity of cellulose. The TGA and DSC data of the PHB_A-04_/ esterified PALF-MCC laurate composites are summarized in Table 2. For the 95:5% (w/w) PHB_A-04_/ esterified PALF-MCC laurate composites, the first weight loss was at 271°C (caused by moisture and volatiles) whereas PHB_A-04_ degradation started at 261°C. It was found that the addition of filler increased the degradation temperature by approximately 10°C for all composites regardless of the fibre concentration [9]. The results indicated that adding cellulose fibre has no effect on the thermal degradation of PHB and P(HB-co-HV) [9, 53]. As shown, all PHB_A-04_/ esterified PALF-MCC laurate composites had 2 degradation temperatures: one is attributed to the slightly decreased T_d_ of the PHB_A-04_ (weight loss more than 80%), and the other is attributed to the T_d_ of esterified PALF-MCC laurate at approximately 355.85°C with a weight loss of 4–15.90% depending on the content of esterified PALF-MCC laurate addition. When fibre was added to biopolymers, the degradation temperature increased marginally according to TGA data [9].

Compared with the DSC thermogram of the neat PHB_A-04_ film, we found that the T_m_ and T_g_ of the composite films did not alter significantly. As listed in the Table 2, a small increase in T_c_ was observed in all PHB_A-04_/ esterified PALF-MCC laurate composites. Although no other endothermic peak was observed for the PHB_A-04_/ esterified PALF-MCC laurate composite films, this does not indicate that the films are compatible. The endothermic transition that occurred in all PHB_A-04_/ esterified PALF-MCC laurate composites is attributed solely to the PHB_A-04_ phase because esterified PALF-MCC laurate, like any other cellulose fibre, has no melting temperature and instead has gelatinization and degradation temperatures [54]. The addition of esterified PALF-MCC laurate did not influence the T_g_ values.

### Effect of the content of esterified PALF-MCC laurate as filler in PHB_A-04_/esterified PALF-MCC laurate biocomposite films on biodegradability

**Fig 11:**
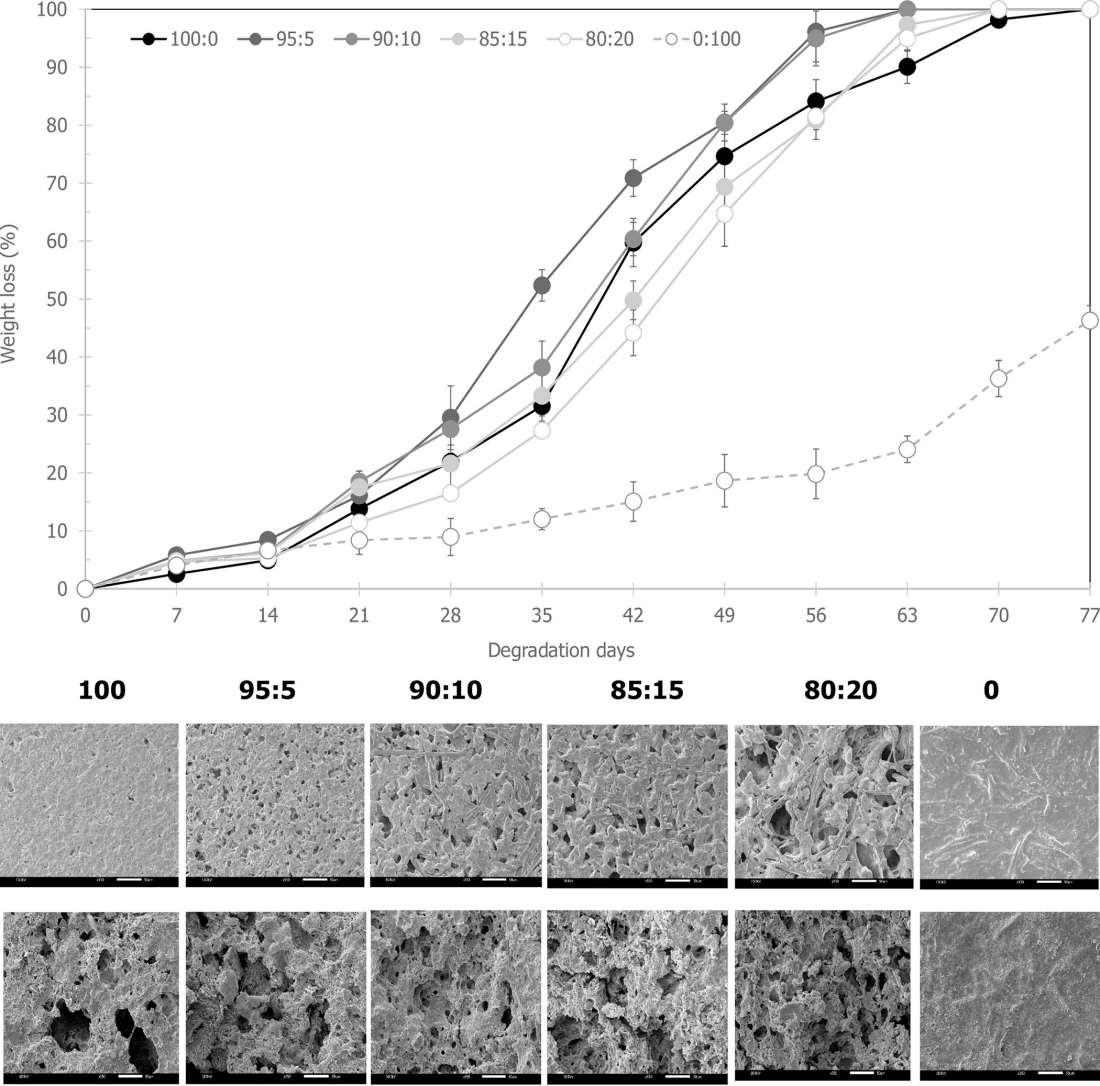
Degradation time courses in laboratory-scale fertilized soil conditions in soil burial test for 77 days (A) % weight loss PHB/esterified PALF-MCC laurate biocomposite films and (B) SEM micrographs of surface morphology of PHB/esterified PALF-MCC laurate biocomposite films before and after 63 days.

The biodegradability of PHB_A-04_/ esterified PALF-MCC laurate biocomposite films was determined in fertilized soil at pH values of 5–7. The soil moisture content was maintained by watering every 24 h to retain 80–100% humidity and temperatures between 20 and 28°C. Samples were removed for weight loss analysis every 7 days. The film surface morphology was investigated by scanning electron microscopy (SEM). The degradation rate was evaluated by determining the weight loss of the film. The linear portion of the curve of the biocomposites is considered by assuming that full degradation has been accomplished. Then, the rate constant, *k*, and half-life of degradation, *T*_1/2_, values were calculated by applying an integrated kinetic equation of the first order rate (Table 3). The initial weight of all samples was approximately 55.7 ± 0.01 mg. The weight loss of the films increased proportionally with degradation time. The main degradation process with a constant surface area is surface erosion [55]. The degradation rate of 1.28% per day was observed for the pure PHB_A-04_ films, and the half-life (*T*_1/2_) was estimated at 39 days. The degradation rate increased as the content of esterified PALF-MCC laurate increased. The pure esterified PALF-MCC laurate film showed the lowest degradation rate of 0.45% per day. Relative acceleration rates were observed when esterified PALF-MCC laurate were added to the PHB_A-04_ matrix. The 95:5% (w/w) PHB_A-04_/esterified PALF-MCC laurate composite films possessing compatibility between the two matrices showed the fastest degradation rate of 1.65% per day because the hydrophilic esterified PALF-MCC laurate enhanced the hydrolysis rate (adsorbed water) while PHB_A-04_ was preferable for degradation. The results indicate that the inclusion of esterified PALF-MCC laurate (slowly degradable) facilitates accelerated degradation of PHB, but at high esterified PALF-MCC laurate loads the change in weight loss becomes more of a function of the esterified PALF-MCC laurate content than the PHB content. Fig 11A illustrates the weight loss of PHB_A-04_ and PHB_A-04_/esterified PALF-MCC laurate films with various amounts of esterified PALF-MCC laurate as a function of the exposure time in soil. Pure esterified PALF-MCC laurate films exposed in soil for 77 days showed slower weight loss than PHB_A-04_, whereas blends with esterified PALF-MCC laurate fibres exhibited greater weight loss than either 100% PHB_A-04_ or 100% esterified PALF-MCC laurate films. For the blends containing 5 and 10% (w/w) esterified PALF-MCC laurate, the weight loss reached 100% at 63 days. It was concluded that the percentage of weight loss increased with the exposure time in the soil as well as the esterified PALF-MCC laurate content. In general, natural fibre-reinforced composites possessed higher degradation rates when they were applied to outdoor applications than composites with synthetic fibres [56]. Biocomposite degradation occurs with the degradation of its individual components and with the loss of interfacial strength between them [57]. The poor interfacial interaction between highly polar natural fibres and a nonpolar matrix might reduce the composite’s final characteristics, limiting its industrial application. In PHB degradation, moisture susceptibility is the initial stage to start the degradation and involves four steps, namely, water absorption, ester cleavage to form oligomers, solubilisation of oligomer fractions, and diffusion of soluble oligomers by soil microorganisms [58]. This study showed that the onset of PHB_A-04_ degradation occurred as early as 7 days and was marked by the deterioration of the samples, resulting in a *T*_1/2_ of 39 days. When compared to pure PHB_A-04_, the degradation of pure esterified PALF-MCC laurate was slower in composites due to the composite’s resistance to water absorption and diffusion. Pure PHB_A-04_ required 39 days to attain *T*_1/2_, whereas pure esterified PALF-MCC laurate required 111 days to reach *T*_1/2_. Fig 11A shows that after 21 days in the fertilized soil, pure PHB_A-04_ and all composites showed marked degradation. At 70 days, all of the PHB_A-04_ and composite samples showed 100% degradation. The degradation parameters shown in Table 3 demonstrate that the PHB_A-04_/esterified PALF-MCC laurate composites have a higher rate of degradation than the pure PHB_A-04_ and esterified PALF-MCC laurate. As a result, PHB_A-04_ and esterified PALF-MCC laurate composites are more water-susceptible and hence more degradable than pure PHB_A-04_ and esterified PALF-MCC laurate composites. It has been well known that the degradation of PHB is promoted by presence of natural fibres [10, 59–61]. The degradation starting with moisture adsorption occurred in the first step before the hydrolysis process [10]. It has been reported that natural fibres facilitate the moisture absorption process whereas PHB is a major substrate for hydrolytic enzymes [60, 62]. Although esterified PALF-MCC laurate showed slow degradation, adding it into the PHB matrix could promote a faster degradation process due to the improvement of water uptake of the composites, and then PHB became susceptible to microbial degradation [60, 62].

**Table 3:** The comparative data of degradation rates of PHB/esterified PALF-MCC laurate biocomposite films with esterified PALF-MCC laurate contents of 0, 5, 10, 15, 20 and 100% (w/w).

Samples	Degradation parameters		
	*k* (% per day)	*T*_1/2_ (day)	Erosion rate [mg (cm^2^ day)^-1^]
**filler**			
esterified PALF-MCC laurate	0.45	111	0.06
**major matrix**			
PHB	1.28	39	0.16
**biocomposite** PHB_A-04_/esterified PALF-MCC laurate			
95:5	1.65	31	0.30
90:10	1.42	32	0.29
85:15	1.34	37	0.24
80:20	1.32	36	0.23

Photographs of PHB_A-04_, esterified PALF-MCC laurate and PHB_A-04_/esterified PALF-MCC laurate composite films containing different amounts of esterified PALF-MCC laurate are shown in Fig 9. After 70 days of exposure, 100% degradation of the pure PHB_A-04_ film was observed. Moreover, the films with 5 and 20% (w/w) esterified PALF-MCC laurate were highly degraded, as observed by the presence of tiny holes on the surface of the films (Fig 11B), which resulted from the various positions on the film surface that could be attacked by microorganisms. This behaviour is caused by the addition of fibres that favour the disintegration of the sample, increasing its susceptibility to microbial attack. These SEM micrographs indicate that PHB_A-04_ is the major carbon source of the bacteria, whereas esterified PALF-MCC laurate is unaffected. As a result of the hydrolysis process, the PHB_A-04_ main chain is scissioned into tiny pieces, which are more vulnerable to microorganism assault. These results were in accordance with the weight loss measurements. Under nonaqueous conditions, PHB_A-04_ degrades by random chain scission, but in the presence of water, PHB_A-04_ degrades via surface hydrolysis with no change in molecular weight [63]. Chen (2009) found that when the film morphology of PHB_A-04_ includes holes on the surface, water molecules can come into contact with the surface, causing the polymer around the holes to breakdown [64]. More bacteria and water molecules can fill the big holes as the pores become larger, causing additional degradation. This procedure would be considerably more difficult on a smooth process surface, as seen in the pure esterified PALF-MCC laurate film.

## Conclusion

The commercialization of PHB has been hampered by its price compared with other bioplastics. In addition, PHB possesses brittleness and thermal instability. There are many reports on blending PHB with other polymers while preserving the renewable, biodegradable and low-cost properties. MCC produced from agricultural waste is considered a non-wood-based resource and well known as a very low price material. The preparation of PALF-MCC and esterified PALF MCC laurate is very simple and cheaper than the PHB production and purification process. This study demonstrates that the addition of low-cost esterified PALF MCC laurate to PHB_A-04_ matrices is able to preserve a satisfactory value for tensile strength, elastic modulus (which decreases with the addition of esterified PALF MCC laurate), and lightly increasing elongation will eventually increase flexibility. The presence of the esterified PALF MCC laurate favoured the disintegration of the sample caused by moisture absorption, increasing its susceptibility to microbial attack, thus resulting in an increase in weight loss.

## Supporting information

S1 Fig Time course photograph of disintegrability of cast PHB/esterified PALF-MCC laurate biocomposite films in laboratory-scale composting conditions in soil burial test for 77 days. (TIF)

## Abbreviations

CDW: cell dry weight
DSC: differential scanning calorimetry
GPC: gel permeation chromatography
PALF: pineapple leaf fibres
PALF-MCC: pineapple leaf fibres-microcrystalline cellulose
M_W_: weight-average molecular weight
ΔH_C_: enthalpy of crystallization
ΔH_M_: enthalpy of fusion
M_N_: the number-average molecular weight
PHB: poly(3-hydroxybutyrate
T_c_: crystallization temperature
T_d_: degradation temperature
T_g_: glass transition temperature
TGA: thermal gravimetric analysis
T_m_: melting temperature
X_C_ (%): percent of crystallinity
