# Supplementary figures and images for: Green composites made of polyhydroxybutyrate and long-chain fatty acid esterified microcrystalline cellulose from pineapple leaf
Source: PLoS One. 2023 Mar 3;18(3):e0282311. doi: 10.1371/journal.pone.0282311 (PMC9983910; doi:10.1371/journal.pone.0282311)

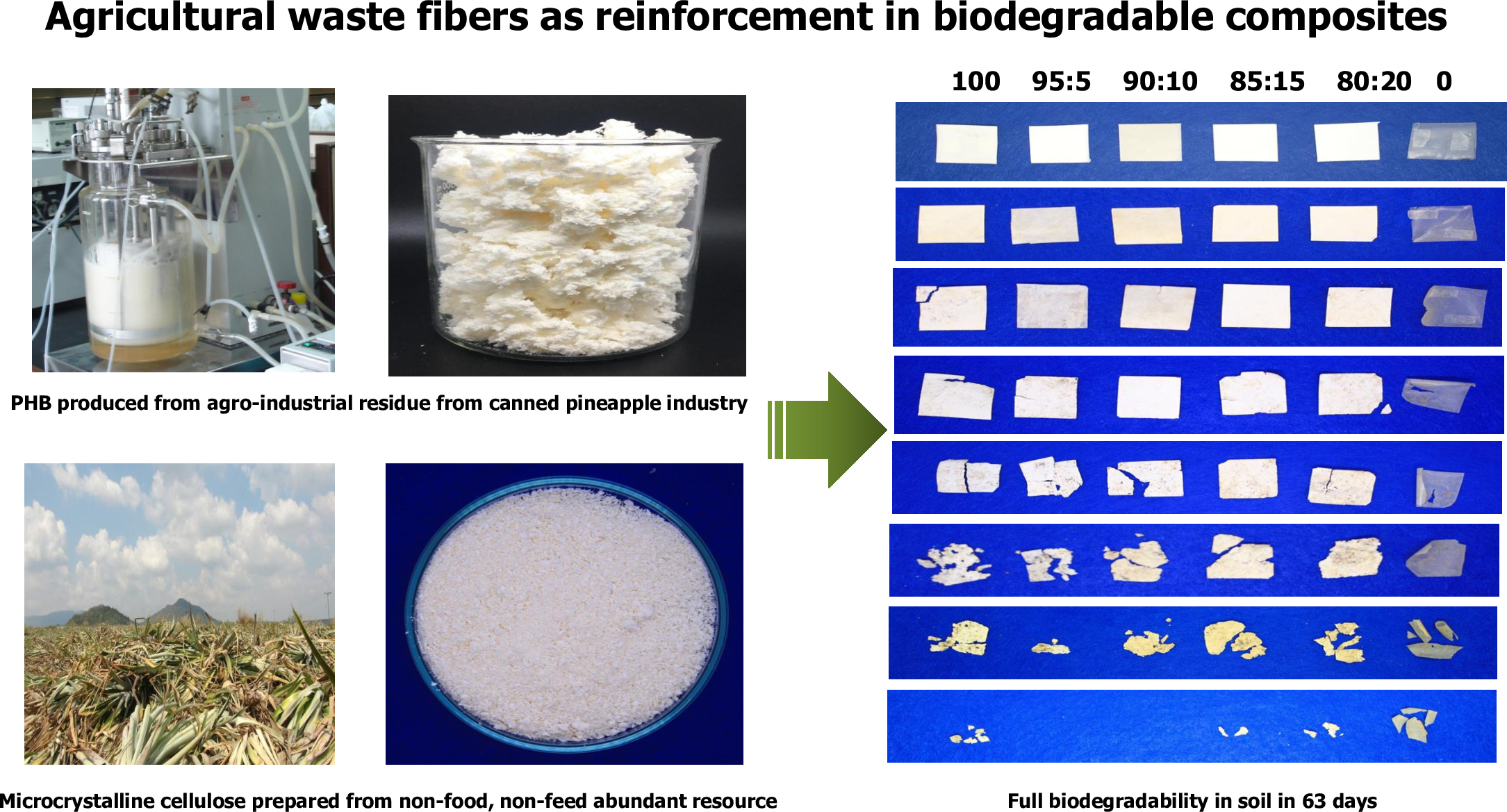

Supplement: S1 Fig — (TIF) [file pone.0282311.s001.tif]
